# Pan-genome of wild and cultivated rice uncovers genetic diversity, lost and selected sequences during rice domestication

**DOI:** 10.1186/s12284-026-00893-w

**Published:** 2026-02-24

**Authors:** Huan Tao, Shuhong Wu, Guofeng Wu, Xuan Chen, Yiqiong Sun, Xiangrong Zhu, Lixin Chen, Changqing Feng, Manegdebwaoga Arthur Fabrice Kabore, Samuel Tareke Woldegiorgis, Lina Zhang, Yufang Ai, Wei Liu, Huaqin He

**Affiliations:** College of Life Sciences, Fujian Agriculture and Forestry University, Fuzhou, 350002 China

**Keywords:** Wild rice, Rice domestication, Pan-genome, Transcriptome, Salt stress

## Abstract

Wild relatives of crops are pivotal genetic reservoirs for enhancing stress resistance and yield of cultivated varieties. Rice (*Oryza sativa* L.) domestication had led to the loss of numerous beneficial traits from wild rice, especially in the AA genome group, the closest wild relatives of cultivated rice. However, existing pan-genome studies of *Oryza* covered only limited set of AA genome wild species or focused on non-AA genome types. In this study, by using genomic re-sequencing data of 180 wild rice varieties, we constructed pan-genomes of five AA genome types including *O. barthii, O. meridionalis, O. glumaepatula, O. longistaminata*, and *O. rufipogon*, in which each comprised 67–224 Mb of novel sequence. The detected genetic variations across these species were substantial, with SNPs ranging from 7.24 × 10^6^ to 2.60 × 10^7^ and InDels from 2.01 × 10^6^ to 7.24 × 10^6^. The results hinted a primary evolutionary gene flowing direction from *O. meridionalis* to *O. rufipogon*, and subsequently to *O. sativa*, reflecting a potential evolutionary trend among AA genome rice species. Notably, several nsSNPs were conserved between *O. meridionalis* and *O. rufipogon*, suggesting their importance throughout the evolutionary process. Many genes related to stress response and growth regulation were found within the lost and selected regions, among which 42 and 32 genes were differentially expressed in *O. rufipogon* and the cultivated variety *Nipponbare* under salt stress. *WRKY67* gene was selected as a strong candidate for salt tolerance of rice, which was further validated by RT-qPCR, co-expression analysis, and phenotypic assessment of *WRKY67* knockout mutant under salt stress. This research has filled the gap in targeted exploration of genetic resources in AA genome rice, and provided the candidate genes directly applicable for salt-tolerant rice breeding.

**Supplementary Information:**

The online version contains supplementary material available at 10.1186/s12284-026-00893-w.

## Introduction

The world’s population is expected to increase by nearly 2 billion persons in the next 30 years, from the current 8 billion to 9.7 billion in 2050 and potentially peaking at nearly 10.4 billion in the mid-2080s (https://www.un.org/en/global-issues/population). To meet the demands of this continued growth, global rice production must increase substantially. Rice holds immense significance in the progress of human civilization and food security. It is a staple food for approximately half of the world's population and contributes to around 50–80% of dietary calories (Tsioumani 2019). However, global rice production is threatened by climate change (Lobell and Gourdji 2012). Throughout the history of rice breeding, exploring new genetic resources and adopting new technologies have become key strategies for making breakthroughs. However, many modern cultivated rice varieties share the same or similar genetic backgrounds, and numerous genes associated with desirable traits have been lost during domestication and intensive breeding. Consequently, the genetic resources available for further improvement are approaching saturation, leading to reduced genetic diversity and diminished resilience against biotic and abiotic stresses. Wild rice has grown in a complex natural environment for a long time, has accumulated a variety of excellent genes that allow it to tolerate many different biotic and abiotic stress conditions (Stein et al. 2018; Atwell et al. 2014; Garg et al. 2013; Mizuta et al. 2010). This genetic reservoir represents a significant untapped resource for crop improvement (Tanksley and McCouch 1997). Previous studies have shown that cultivated rice (*Oryza sativa*) possesses only about 60% of the alleles found in its wild relatives, and that the population structure of *O. rufipogon* exhibits far greater genetic diversity than that of *O. sativa* (Sun et al. 2001, 2002). Harnessing the exceptional genes present in wild rice is crucial to overcome the genetic uniformity of cultivated varieties. Moreover, future breeding must equip new rice varieties with resilience to environmental stresses and resistance to diseases and pests.

Rice plays a vital role in global agriculture, yet its evolutionary history particularly the relationships between cultivated and wild species remains incompletely resolved. The genus *Oryza* comprises of the two domesticated taxa (*O. sativa* and *O. glaberrima*) and 25 wild species. Among these, 16 wild species are diploid and have been categorized into six genome types (AA, BB, CC, EE, FF, and GG). The 6 wild rice species, *Oryza rufipogon*, *Oryza nivara*, *Oryza meridionalis*, *Oryza barthii*, *Oryza glumaepatula* and *Oryza longistaminata* are AA genome type. The remaining nine species belong to five tetraploid species types (BBCC, CCDD, HHJJ, HHKK, and KKLL) (Fornasiero et al. 2022; Solis et al. 2020). There are views that the cultivated species in the AA genome originated from a common ancestor, and Asian and African cultivated rice was considered to have undergone parallel evolution (Khush 1997). *O. rufipogon* is considered the ancestor of Asian cultivated rice (Zheng and Ge 2010), while African cultivated rice was independently domesticated from *O. barthii* (Wang et al. 2014). Among the eight AA-genome rice species, which one represents the most basal ancestral species remains a long-standing scientific question. Most analyses distinguish *O. meridionalis* and *O. longistaminata* from other AA-genome species, identifying them as the earliest-diverging lineages. However, a consensus on the most basal ancestor is lacking: some studies suggest it is *O. meridionalis*, while others propose *O. longistaminata* (Zhu and Ge 2005; Zou et al. 2008; Ren et al. 2003; Wambugu et al. 2015). The parallel and independent origins of African and Asian cultivated rice, as well as the evolutionary relationships between their wild ancestral species and other AA genotype rice species, are worthy of further exploration.

Rice plants grow in dynamic environments and frequently experience various abiotic stresses, such as drought, high salinity, cold, and heat (Zhu 2016). Soil salinity is one of the most severe abiotic stresses affecting rice growth and productivity (Hoang et al. 2016). Rice is a salt-sensitive crop (Chinnusamy et al. 2005), salinity stress suppresses photosynthesis and growth, leading to biomass loss, as well as partial sterility, which ultimately results in reductions in rice yield, especially during the seedling stage. Consequently, developing salt-tolerant varieties represents the most economical and effective strategy to sustain and expand rice production in saline-alkali soils (Qin et al. 2020). Yuan et al. analyzed salt tolerance using a backcross inbred line (BIL) population derived from a cross between indica rice 9311 and *O. longistaminata*. They identified 27 quantitative trait loci (QTLs) associated with salt tolerance. Further sequencing and expression analysis revealed that a cytochrome gene, *MH02t0466900*, was a key candidate gene related to salt tolerance (Yuan et al. 2022). In other study, Quan et al*.* used salt-tolerant wild rice Dongxiang and the cultivated rice variety NJ16 as materials, and co-localized nine quantitative trait loci (QTLs) for seedling-stage salt tolerance. Among these, *qST1.2* and *qST6* were identified as major-effect loci capable of enhancing salt tolerance in rice (Quan et al. 2018).

With the continuous development of genome sequencing technology, the decoding of the whole genome of numerous species has been accomplished. It is of paramount importance to investigate the location of functional genes based on a comprehensive analysis of genome information. But, solely relying on the reference genome of a single individual to study genetic domestication and variation might result in the loss of valuable genetic code information. This is because certain individual-specific genome sequences may not be incorporated in the reference genome. Recent research has revealed that the rice reference genome *Nipponbare* lacks many functional genes specific to other rice varieties. For instance, GW5, a gene associated with grain width, and Sub1A, a gene related to submergence tolerance, are both present in rice pan-genomes but absent in *Nipponbare* (Yao et al. 2015; Weng et al. 2008; Xu et al. 2006). Therefore, the construction of a rice pan-genome is crucial for studying rice genetic diversity. It is especially important to construct pan-genomes that cover different stages of domestication, regions, and genomic types of rice varieties. This will greatly contribute to our understanding of the evolution and domestication process of rice. At present, pan-genome studies have been conducted in over 20 plant species, such as corn (Hirsch et al. 2014), soybean (Li et al. 2014), tomato (Gao et al. 2019), and cotton (Li et al. 2021). In rice, several pan-genome studies have been reported, including two studies constructed a pan-genome using short reads of 453 *O. sativa* accessions, 53 *O. sativa* and 13 *O. rufipogon* accessions (Wang et al. 2018; Zhao et al. 2018), three other studies constructed pan-genomes using long genome re-sequencing reads (Qin et al. 2021; Zhang et al. 2022a; Shang et al. 2022). Whilst, 145 chromosome-level genome re-sequencing reads were used to develop a rice pan-genome (Guo et al. 2025). However, these pan-genomes primarily focused on the domestication of *O. sativa*, and many wild rice accessions have not been fully explored, especially the AA-genome wild rice varieties.

In this study, we used genome resequencing data of 180 wild and 39 cultivated rice accessions to investigate rice domestication. We analyzed evolutionary gene flow in cultivated rice and identified sequences lost and selected during its evolution. Furthermore, we screened for differentially expressed genes within these lost and selected regions in both wild and cultivated rice under salt stress. This work not only fills a critical gap in exploring AA-genome rice genetic resources but also provides candidate genes directly applicable to salt-tolerant rice breeding.

## Results

### Pan-genome of wild rice

**Fig. 1:**
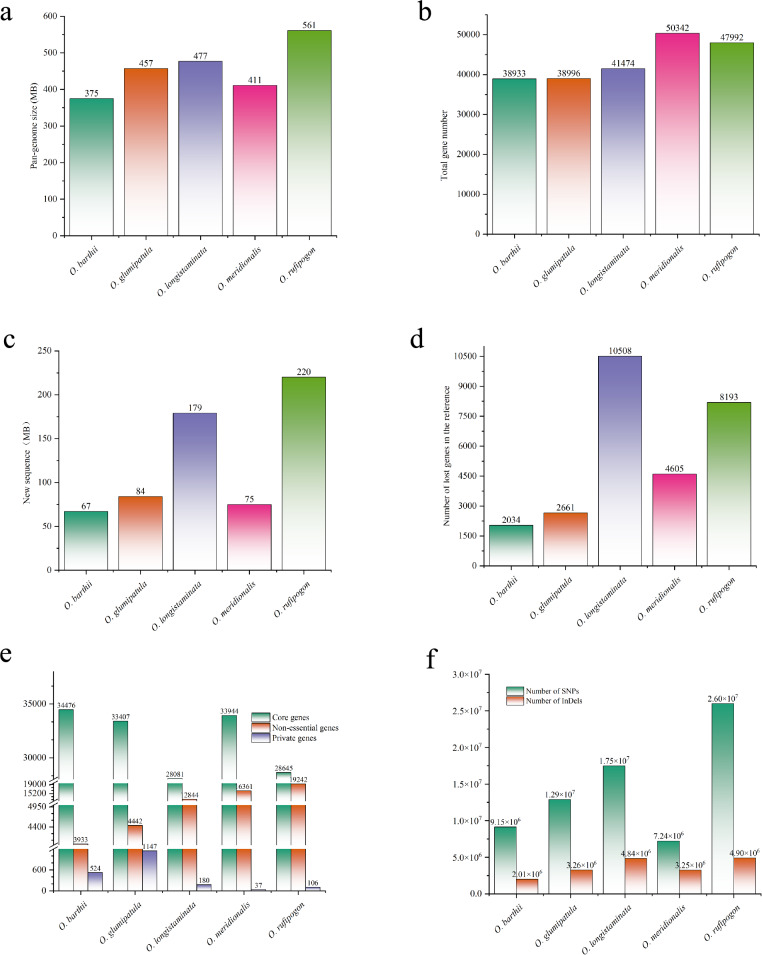
Basic information of 5 wild rice pan-genomes. **a** The sizes of the pan-genomes of the five wild rices (Mb). **b** The number of genes included in the pan-genomes of the five wild rices. **c** The non-redundant novel sequences in the pan-genomes of the five wild rices. **d** The number of genes lost in the reference genomes of the five wild rices. **e** The number of core genes, non-essential genes, and private genes in the pan-genomes of the five wild rices. **f** The number of SNPs and InDels in the five wild rices

The genome re-sequencing data of 180 wild rice varieties, including 20 *O. barthii*, 26 *O. meridionalis*, 21 *O. glumaepatula*, 24 *O. longistaminata*, 89 *O. rufipogon* and 39 *O. sativa* varieties were collected (Table S1, S2). The average sequencing depth of all samples was higher than 14 × . Using an iterative assembly strategy, we constructed pan-genomes for the five AA-genome wild rice species (*O. barthii*, *O. meridionalis*, *O. glumaepatula*, *O. longistaminata*, *O. rufipogon*). Assembly quality was assessed with the BUSCO tool. Over 95.0% of conserved plant genes were detected in the pan-genomes of four species (*O. barthii*, *O. meridionalis*, *O. glumaepatula*, *O. rufipogon*), whereas only 1,445 (89.5%) were found in *O. longistaminata* (Figure S1). The pan-genome sizes ranged from 375 to 561 Mb (Fig. 1a), containing 38,933 to 50,342 genes (Fig. 1b) and 67–220 Mb of non-redundant novel sequence (Fig. 1c). Additionally, 2034 to 10,508 genes present in the pan-genomes were absent from the respective reference genomes (Fig. 1d). A total of 1994 genes were identified across the five wild rice pan-genomes, each present in only a single sample (Fig. 1e).

### Variation in the pan-genome of wild rice

Using cultivated rice *Nipponbare* as the reference genome, the average mapping rates of *O. barthii*, *O. meridionalis*, *O. glumaepatula*, *O. longistaminata* and *O. rufipogon* were 95.99%, 95.25%, 95.86%, 95.24% and 96.31%, respectively, with corresponding genome coverage values of 83.98%, 77.83%, 81.53%, 77.51%, and 90.22%. The number of SNPs detected in each wild rice species ranged from 7.24 × 10⁶ to 2.60 × 10⁷, while the number of InDels ranged from 2.01 × 10⁶ to 4.90 × 10⁶. (Fig. 1f). In addition to these small variants (variation length < 50 bp), abundant structural variations (SV) were identified in wild rice, including 614,365 InDels (variation length ≥ 50 bp) with a total length of 34.05 Mb, as well as frequent inversion and translocation variations. Notably, *O. longistaminata* harbored significantly more translocations (12,414 variants; 91.79 Mb) than other species (Table S4, S5).

The presence and absence of genes (PAV genes), a type of genomic structural variation, was an index to show the percentage of genes present only in a single sample or partial samples. Generally, PAV gene is associated with plant adaptation to environment or unique biological characteristics, reflecting breed specificity. We detected 4,457 to 19,348 PAV genes in the five wild rice pan-genomes. *O. rufipogon* possessed the highest total number of PAV genes, whereas *O. glumaepatula* contained the most private genes (genes unique to a single accession) (Fig. 1e, Table S6). GO enrichment analysis of these PAV genes revealed that the top 15 significantly enriched GO terms were predominantly associated with response to stress (Table S7). The terms response to chemical and response to endogenous stimulus were enriched across four wild rice species (*O. barthii*, *O. longistaminata*, *O. meridionalis* and *O. rufipogon*). These results collectively indicated that PAV genes in wild rice exhibit a distinct functional bias toward environmental adaptation.

### Phylogenetics of the cultivated and wild rice

**Fig. 2:**
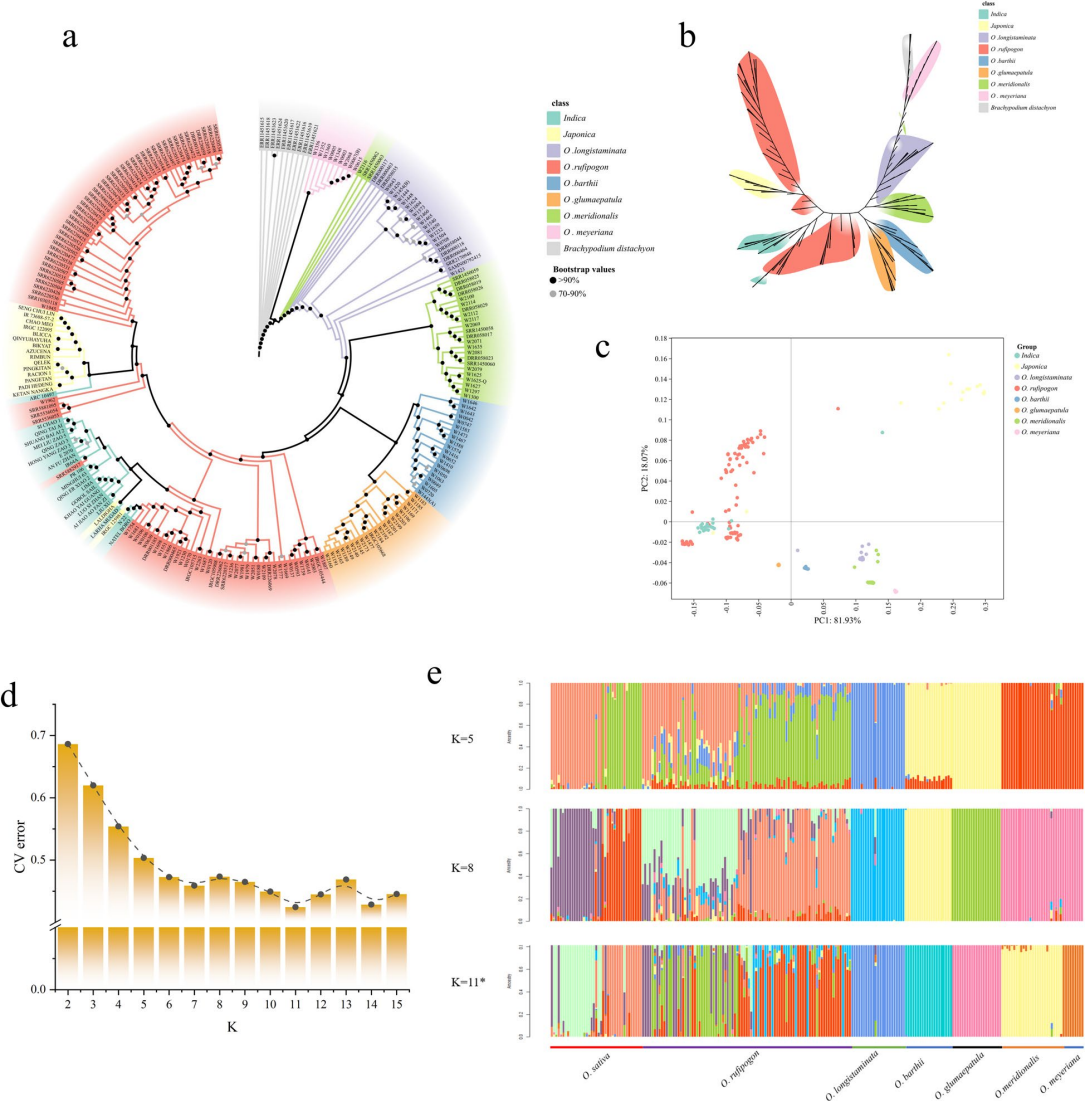
Phylogenetics and population structure of wild and cultivated rice. **a** Circular phylogenetics tree of wild and cultivated rice. **b** Unrooted phylogenetic tree of wild and cultivated rice. **c** PCA analysis of wild and cultivated rice. **d** The CV error When k value from 2 to 15. **e** Population structure of rice varieties tested at K = 5, 8 and 11. Different rice subspecies were sorted, and "*" indicated the best grouping number with the minimum cross-validation error

Previous studies have suggested that *O. meyeriana* was the ancestor of wild rice. To root our phylogenetic analysis, we therefore included nine *O. meyeriana* and ten *Brachypodium distachyon* samples as outgroups. After stringent filtering, we identified a set of 546,380 high-quality SNPs. A maximum-likelihood phylogenetic tree constructed from these data showed that four of the five AA-genome wild species (*O. barthii, O. meridionalis, O. glumaepatula*, and *O. longistaminata*) formed distinct, species-specific clades. The two subspecies of cultivated rice, *Japonica* and *Indica*, were also grouped into one family. *O. rufipogon* was divided into three branches, two of which were clustered with *Japonica* and *Indica* rice (Fig. 2a, b). *O. barthii* and *O. glumaepatula* were clustered together. *O. meridionalis* exhibited the shortest evolutionary distance to *O. rufipogon*. Principal component analysis (PCA) supported these phylogenetic relationships (Fig. 2c). PCA clearly separated samples by subspecies, with *japonica* and *indica* varieties forming distinct clusters. Most *O. rufipogon* accessions were positioned intermediately between the two cultivated groups, consistent with its ancestral and admixed phylogenetic position.

To determine the optimal population structure, we calculated the cross-validation error (CV values) from K = 1 to 15. The CV error reached a minimum (0.42439) at K = 11, identifying this as the most suitable model for population subdivision (Fig. 2d). Under K = 11, cultivated rice accessions were primarily composed of two ancestral components corresponding to the *japonica* and *indica* subspecies. On the other hand, *O. rufipogon* exhibited a complex, admixed ancestry, reflecting extensive historical introgression*.* In contrast the other 5 wild rice subspecies, except for the infiltration of gene sequences of other subspecies in few samples, all had a unique ancestral composition. At K = 8, the ancestral components subdivided further, separating the original dataset into eight distinct clusters: six corresponding to wild species and two to the *japonica* and *indica* cultivated groups. At this level, *O. rufipogon* genotype groups were more clearly separated, some of which were similar to the cultivated rice. *O. meridionalis* and the outgroup *O. meyeriana* shared similar ancestral components, indicating a close phylogenetic relationship. The rice accessions in this study were collected from 5 regions, Africa, Asia, North America, South America and Oceania. When K = 5, the population clusters aligned strongly with these geographic origins, allowing for clear identification the origin of rice (Fig. 2e).

**Fig. 3:**
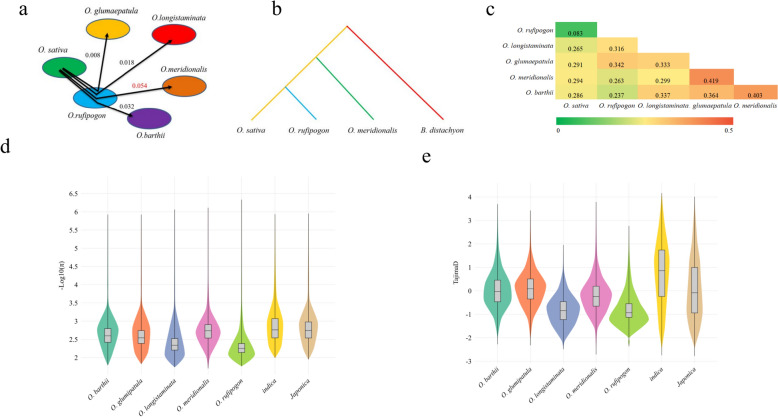
ABBA-BABA test and Genetic diversity of wild and cultivated rice. **a** ABBA-BABA test analysis between wild and cultivated rice. **b** Gene flow in the evolution of cultivated rice. **c** Fixation index analysis of wild and cultivated rice. **d** Nucleotide diversity in wild and cultivated rice. **e** Tajima's D statistics of wild rice and cultivated rice

We performed ABBA-BABA tests (D-statistics) using the following topology: *Brachypodium distachyon* as outgroup, *O. sativa* as P1, *O. rufipogon* as P2, and other AA genome wild rice as P3 (Heliconius Genome 2012). The highest D value of 0.054 was observed between the *O. rufipogon* and *O. meridionalis* group (Fig. 3a),indicating that the main direction of gene flow relevant to the origin and domestication of *O. sativa* is likely from *O. meridionalis* to *O. rufipogon*, and then to *O. sativa* (Fig. 3b). This inference was based on current sample and genetic data, and did not exclude other potential minor gene flow events or complex evolutionary scenarios (Fig. 3b).

### Genetic diversity of wild and cultivated rice.

To characterize genomic variation, population structure, and evolutionary dynamics in wild rice, we evaluated the genetic diversity between cultivated and wild rice by using nucleotide diversity (π), Tajima's D statistics, and fixation index (FST). *O. rufipogon* showed the highest nucleotide diversity of 0.00568, followed by *O. longistaminata* of 0.00476 (Fig. 3d). The genetic diversity of cultivated rice (*indica* and *japonica*) was significantly lower than that of wild rice, indicating the effects of genetic bottlenecks during rice domestication. Most wild rice (*O. longistaminata*, *O. rufipogon*, *O. meridionalis*) exhibited negative Tajima’s D values, indicating that these populations might have undergone recent population expansion or been subject to positive selection. In contrast, *O. barthii* and *O. glumipatula* had Tajima’s D values close to zero or slightly positive, which suggested that their populations were closer to the neutral evolutionary equilibrium or experienced balancing selection to maintain the polymorphism of key loci. In striking contrast, indica rice showed a significantly positive Tajima’s D value, implying that it had undergone more intense artificial selection (Fig. 3e). *O. rufipogon* and *O. sativa* had the smallest FST of 0.083, indicating that there was the lowest genetic differentiation between *O. rufipogon* and *O. sativa* (Fig. 3c).

### Lost and selected genes in the cultivated rice

**Fig. 4:**
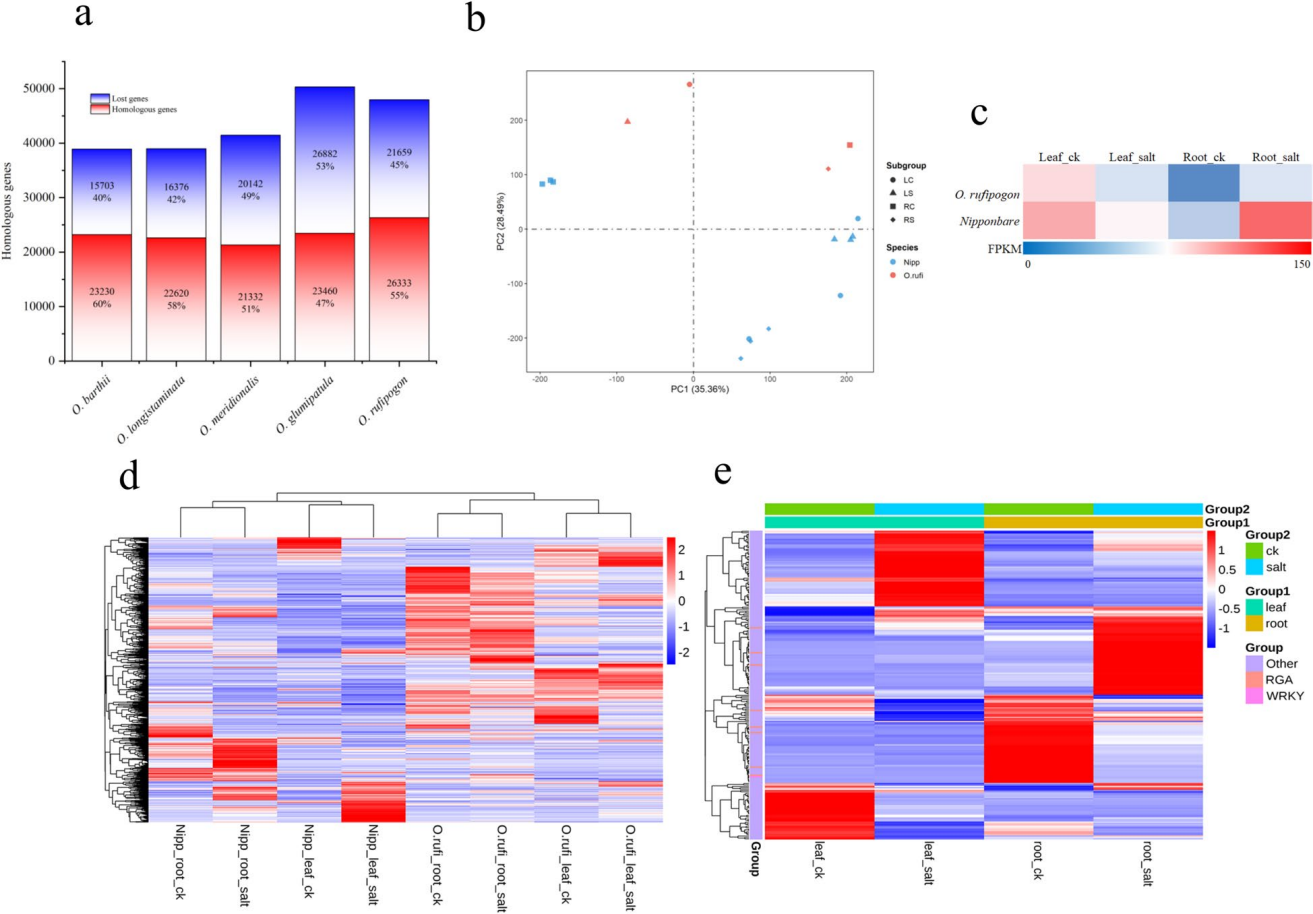
**a** Homologous and lost genes of wild rice. **b** PCA of Replicate Samples from RNA-Seq. LC, Leaf _CK; LS, Leaf_salt; RC, Root_CK; RS, Root_salt. Wild rice samples had no biological replicates, whereas cultivated rice samples had three. **c** Expression pattern of *WRKY67*. **d** Differential expression heat map of 1139 selected genes in the evolution of *O. rufipogon* to *O. sativa*. FPKM of 1139 selected genes in the leaves and roots of *O. rufipogon* and *O. sativa* under salt stress were normalized and heat maps were drawn. **e** Heat map of differential expression gene in lost gene set in leaves and roots of *O. rufipogon* under salt stress. FPKM of differential expression gene in lost gene set in the leaves and roots of *O. rufipogon* was normalized to draw heat map

Using homology alignment against the wild rice pan-genome, we identified sets of homologous genes shared between wild and cultivated rice. During the domestication transition from wild progenitors to cultivated *O. sativa*, a substantial number of genes were lost, ranging from 15,730 to 26,882 across comparisons. *O. glumaepatula* showed a higher tendency for gene loss compared to other wild species (Fig. 4a). The percentage of PAV genes in homologous gene sets and lost gene sets of *O. longistaminata* and *O. rufipogon* was higher than that of other wild rice species, except *O. meridionalis*. This result suggested PAV gene gain and loss happened more frequently in *O. longistaminata* and *O. rufipogon* (Table S8, S9). Previous studies found that the protein involving interaction networks tended to be more resistant to loss during evolution than that outside the interaction networks (Bayer et al. 2021). We analyzed the relationship between PAV status and protein interaction network. In the 5 AA wild rice pan-genomes, 82.4% of the core genes and 44.8% of the PAV genes existed in the protein interaction network (Table S10). The results showed that in the process of evolution from wild rice to cultivated rice, PAV genes were more likely to be lost.

To identify genomic regions under selection during rice evolution, we performed selective sweeps by using Fst and XP-CLR. A total of 190, 64 and 1139 selected genes were identified in the evolutionary transtion from *O. meridionalis* to *O. rufipogon*, *O. meridionalis* to *O. sativa* and *O. meridionalis* to *O. sativa*, respectively (Figures S2–5)*.* GO enrichment analysis of the selected genes indicated that most of the genes are associated to stress response or growth regulation (Figures S6–8, Tables S11–13). From the gene flow path leading to *O. sativa*, we identified five high-confidence selected genes with known functions (Table S14)*,* which are likely involved in growth, development, and stress resistance. These five genes harbored a substantial number of nonsynonymous SNPs (nsSNPs) in wild rice. Further analysis of the genotypes of nsSNPs that located in the same loci in *O. meridionalis* and *O. rufipogon*, it was found that except for heterozygous mutations at very few sites, most of the nsSNPs had the same genotype in *O. meridionalis* and *O. rufipogon*. These results showed that the nsSNPs of these same loci were retained in the evolution from *O. meridionalis* to *O. rufipogon*. If these nsSNPs affected the gene function, the function of these genes was preserved in *O. rufipogon* during the evolution from *O. meridionalis* to *O. rufipogon*, but changed in *O. sativa* during the evolution from *O. rufipogon* to *O. sativa.*

### Identification of candidate genes related to salt stress

*O. rufipogon* harbors various desirable traits, such as resistance to blast, drought tolerance, cold tolerance, and salt tolerance. During the evolution of *O. rufipogon* to cultivated rice, some of superior trait genes were selectively fixed in cultivated rice, while others were lost. Identifying the superior trait genes from wild rice and introgressing them to the improvement of new cultivated rice varieties is an urgent issue in current rice research. This study focuses on the identification of genes related to salt stress tolerance. Thus, using the transcriptome data of *O. rufipogon* and *Nipponbare* under three days of salt stress, the candidate genes in the selected and lost gene set for salt tolerance were identified.

After 3 days of salt stress, the leaves and roots of *O. rufipogon* and *O. sativa* were collected for transcriptome sequencing. *Nipponbare* was used as reference genome for transcriptome analysis. The results of PCA showed that the first two principal components collectively explained 63.85% of the total transcriptome variation. Among them, PC1 (35.36%) primarily separated the species differences between wild rice and *Nipponbare*, while PC2 (28.49%) mainly distinguished the tissue differences between leaves and roots (Fig. 4b). The effect of salt treatment on the transcriptome was smaller than the inherent differences between species and tissues, and the consistent clustering of replicate samples indicated good experimental reproducibility. A total of 313 and 282 differentially expressed genes were identified in the evolutionary selected regions of the leaves and roots of *O. sativa*, while 194 and 160 differentially expressed genes in the leaves and roots of *O. rufipogon*, respectively (Fig. 4d). These DEGs were further filtered based on functional annotation and a stringent expression fold change threshold (|log₂FC|≥ 5). This yielded a final set of 42 high-confidence candidate genes, including 19 resistance gene analogs (RGAs) and the WRKY transcription factor *WRKY67* (Table S15).

**Fig. 5:**
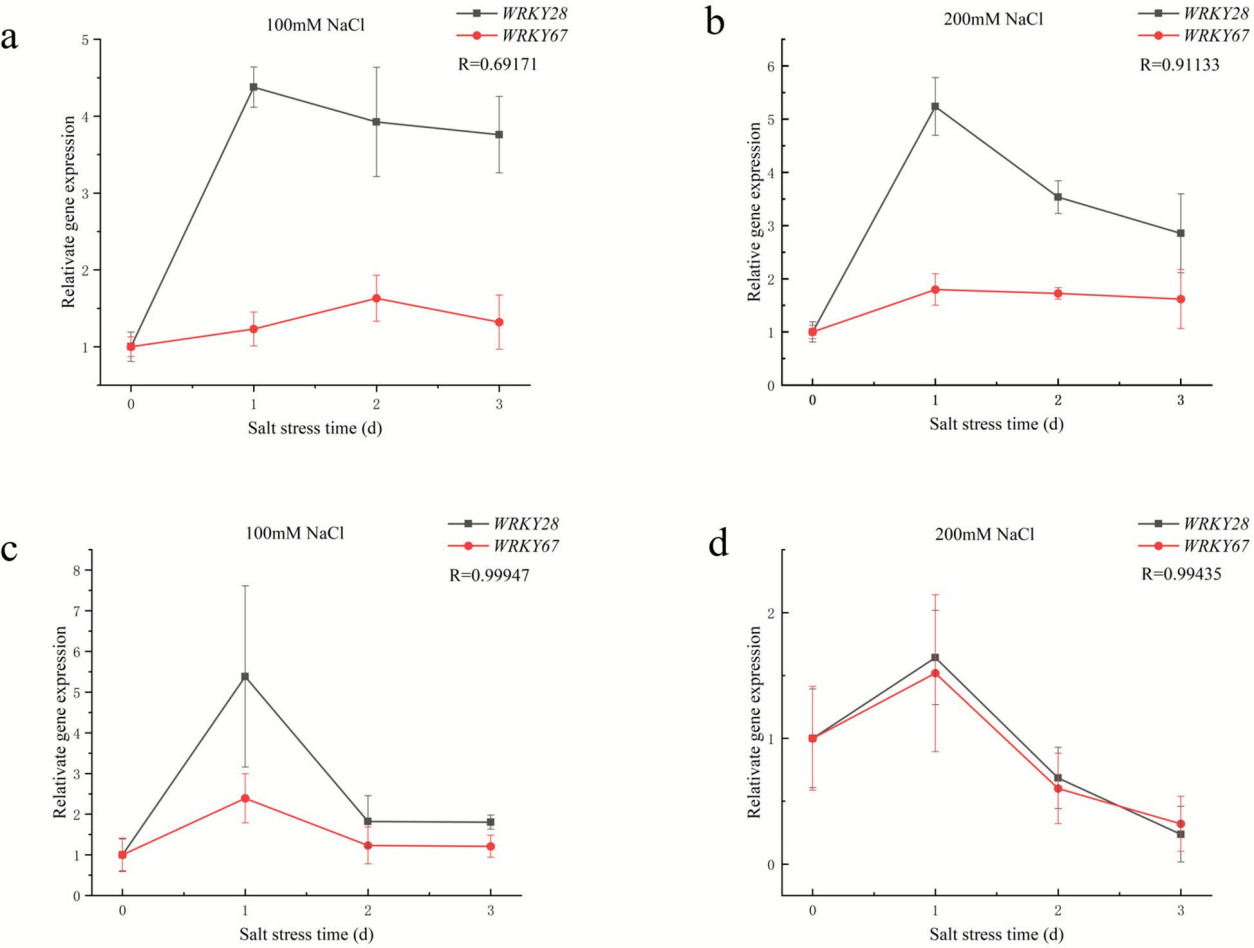
RT-qPCR results of *WRKY28* and *WRKY67* in the leaves and roots of cultivated rice after 1, 2 and 3 days of incubation with 100 mM and 200 mM NaCl. The experiment was performed with 3 biological replicates, and error bars represent the standard deviation (SD). **a** RT-qPCR of *WRKY28* and *WRKY67* in the leaves of cultivated rice under 100.0 mM NaCl stress. **b** RT-qPCR *WRKY28* and *WRKY67* in the leaves of cultivated rice under 200.0 mM NaCl stress. **c** RT-qPCR of *WRKY28* and *WRKY67* in the root of cultivated rice under 100.0 mM NaCl stress. **d** RT-qPCR of *WRKY28* and *WRKY67* in the root of cultivated rice under 200.0 mM NaCl stress. R value represents Pearson Correlation Coefficient

Using the *O. rufipogon* pan-genome as reference for transcriptome analysis, 181 and 183 lost genes were differentially expressed in leaves and roots of *O. rufipogon*, respectively (Fig. 4e). Use the similar criteria, 32 genes including 11 RGAs and 1 WRKY transcription factor genes were identified (Table S16, S17). Among these genes, *WRKY67* was selected for functional validation using the RT-qPCR method. This gene was differentially up-regulated in both the leaves and roots of *Nipponbare*, and in the roots of *O. rufipogon* (Fig. 4c). The known salt tolerance gene *WRKY28* was used as a marker. RT-qPCR results showed that the expression of *WRKY67* and *WRKY28* was up-regulated in the leaves and down-regulated in the roots of cultivated rice, which was consistent with the results of transcriptome analysis. These results indicated that *WRKY67* was induced by salt stress. Under 100 mM and 200 mM NaCl stress, the expression levels of *WRKY67* and *WRKY28* were strongly correlated in both leaves (Pearson’s correlation = 0.69 and 0.91, respectively) and roots (Pearson’s correlation = 0.9975 and 0.9947, respectively) (Fig. 5). These results suggested that these 2 WRKY transcription factors had similar expression trends in the cultivated rice in response to salt stress.

**Fig. 6:**
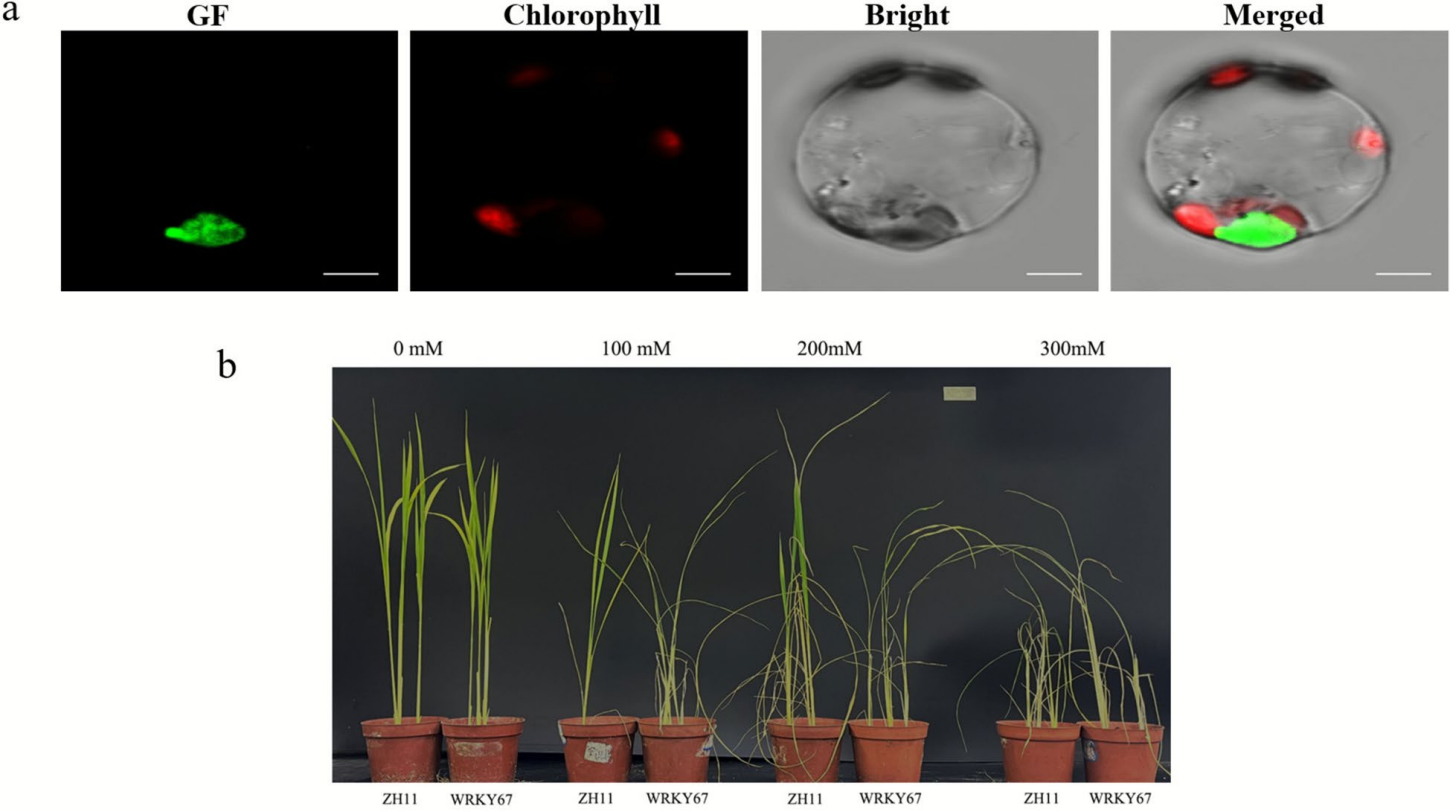
Subcellular Localization of *WRKY67* gene product and phenotypes of WT and WRKY67-knockout mutant under Salt Stress. **a** *WRKY67* encodes a protein is localized in the nucleus. Subcellular localization of *WRKY67* in rice protoplasts. GFP, green fluorescent protein. Bar = 10 μm. **b** After 5 days of salt stress at concentrations of 0/100/200/300 mM NaCl, the phenotypes of young rice seedlings of ZH11 and its *WRKY67* mutant were observed

The prediction software TargetP (https://services.healthtech.dtu.dk/services/TargetP-2.0/) and CELLO (http://cello.life.nctu.edu.tw/) were used to predict the localization of *WRKY67*, and both software results showed that the protein product of *WRKY67* gene was localized in the nucleus. To further validate this result, the full-length CDS of *WRKY67* was constructed into a PGH-GFP expression vector and fused with a 35 s promoter-driven GFP tag. This construct was then transformed into rice stem protoplasts for transient expression. Further repeated experiments showed that the GFP signal from the fusion protein *WRKY67*-GFP was expressed in the nucleus (Fig. 6a). These results demonstrated that the protein product of *WRKY67* gene was localized in the nucleus.

To further verify the salt stress tolerance function of *WRKY67* gene, the rice seedlings of wildtype (WT) and the *WKRY67-knockout* mutant were grown in the nutrient solutions with different salt concentrations. After 5 days of salt stress treatment, phenotypic observations were made. The results indicated that without salt treatment (0 mM NaCl), both WT and *WRKY67-knockout* mutant rice seedlings grew normally. However, under 100 mM and 200 mM NaCl treatment, WT rice seedlings exhibited better growth than the *WRKY67-knockout* mutant. However, under 300 mM NaCl treatment, both WT and *WRKY67-knockout* mutant rice plants showed very diminished growth (Fig. 6b). These results collectively suggested that *WRKY67* was a novel salt-tolerant gene in rice.

## Discussion

The reference genomes of most crops are derived from cultivated varieties, which inevitably fail to capture the full spectrum of genetic diversity present in their wild relatives. This limitation constrains in-depth studies of crop evolution and domestication history (Olsen and Wendel 2013; Meyer and Purugganan 2013). Pan-genome analysis has therefore become essential to overcome this single-reference bias. Wang et al. identified more than 10,000 novel full-length protein-coding genes and a high number of presence-absence variations by using rice pan-genome methods (Wang et al. 2018), while Zhao et al. also found that a total of 10,872 genes in 67 rice accessions were partially absent in the reference genome *Nipponbare* (Zhao et al. 2018). Previous rice pan-genome studies mostly used cultivated rice and a small number of wild rice relatives, or wild rice of different genome types, to construct pan-genomes. Long et al. study covered 7 genome types, with limited depth in AA genome-specific traits (Long et al. 2024). The study by Guo et al. included 16 cultivated rice varieties, diluting the focus on AA genome wild rice (Guo et al. 2025). In this study, we focused on analyzing AA genome wild rice and constructed pan-genomes for 5 AA genome wild rice accessions using short reads genome re-sequencing data from 180 accessions. These pan-genomes contain 67 MB to 224 MB of non-redundant novel sequences. Notably, the BUSCO completeness of the *O. longistaminata* pan-genome was only 89.5%, which was substantially lower than that of the other four AA-genome wild rice species (all > 95.0%). *O. longistaminata* exhibited extensive structural variations, including 12,414 translocation variants, which could disrupt the continuity of conserved orthologous sequences, thereby compromising the accuracy of gene annotation (Goel et al. 2019). Furthermore, the protracted adaptive evolution of this species in its specific habitats might have driven the divergence or even loss of partial BUSCO core genes, thus leading to potential taxonomic bias in BUSCO core gene detection. We found that 1994 genes were private to a single accession. Using *Nipponbare* as the reference genome, a total of 7.24 × 10^6^ to 2.60 × 10^7^ SNPs and 2.01 × 10^6^ to 4.90 × 10^6^ InDels were detected, as well as large number of structural variations (SVs), especially frequent inversion and translocation mutations.

As a major class of structural genomic variation, presence-absence variations (PAVs) contribute to intraspecific individual differences. The dispensable genes carried by individuals may underpin functional significance at the individual or population level, potentially mediating phenotypic diversity and adaptive traits within species. (Rosani et al. 2024). Previous studies had shown that only 53.5 to 62.3% of *O. sativa* genome was consensus sequences, revealing widespread presence and absence variation in *O. sativa* (Hu et al. 2018). Abdullah et al*.* completed de novo chromosome-level assemblies of Australian *O. rufipogon*-like taxon and *O. meridionalis*, identified 320 *O. meridionalis*-specific genes, of which 15% were related to disease resistance and 10% were associated with abiotic stress tolerance (Abdullah et al. 2025). In this study, 4457 to 19,348 PAV genes were detected in the pan-genomes of 5 wild rice. GO enrichment analysis of PAV genes in these 5 wild rice genomes showed that most of the first 15 enriched GO items were related to various stress responses. Tian et al. also found that the PAV genes in plant pan-genomes were related to various stress responses (Tian et al. 2003). The PAV of disease resistant gene had been observed in monocotyledons and dicotyledons, as well as human pan-genomes (Golicz et al. 2016; Hurgobin et al. 2018; Gao et al. 2019; Kehr et al. 2017). Analysis of the relationship between PAV genes and protein–protein interaction networks revealed a potential trend toward increasing PAV gene loss in cultivated rice during domestication. Collectively, these findings support the hypothesis that PAVs from wild rice represent a valuable reservoir of genetic variation that can be harnessed to breed cultivars with enhanced resilience to environmental stresses.

Phylogenetic analysis of based on population SNP data showed that *O. rufipogon* was clustered into three distinct clades. Consistent with previous studies (Huang et al. 2012; Guo et al. 2025), these clades correspond to Or-I, Or-II, and Or-III, where Or-I and Or-III were identified as the ancestral lineages of *Indica* and *Japonica* rice, respectively (Fig. 2a). *Indica* and *Japonica* rice clustered together with Or-I and Or-III, respectively, supporting the hypothesis that *O. rufipogon* was the direct progenitor of *O. sativa* (Second 1982; Wang et al. 1992). However, some other previous studies utilizing nuclear gene sequencing and MITE insertion introns as markers proposed *O. meridionalis* as the earliest diverging lineage (Zhu and Ge 2005; Zou et al. 2008), others supported *O. longistaminata* as the most ancestral species, while the AA genome rice lineage originating from Africa (Ren et al. 2003; Wambugu et al. 2015). Our phylogenetic analysis indicated that *O. longistaminata* was more closely related to *O. meyeriana* than to other taxa, except for three *O. meridionalis* samples. The gene flow analysis results showed that the primary direction of evolutionary gene flow was likely from *O. meridionalis* to *O. rufipogon*, and subsequently to *O. sativa*. It should be noted that rice evolution involves complex geographic dispersal, interspecific gene flow, and artificial selection intervention. The genetic analysis of AA genome wild rice samples in this study revealed the major directions of gene flow and potential evolutionary trends, rather than an exclusive evolutionary pathway.

In this study, we characterized the genetic differences between wild rice and cultivated rice using nucleotide diversity (π), Tajima's D statistic, and fixation index (FST), providing molecular genetic evidence for research on rice domestication and the adaptive evolution of wild rice. The nucleotide diversity of wild rice was significantly higher than that of cultivated rice (*indica* and *japonica*), which further confirmed that the genetic bottleneck effect is a core feature of rice domestication(Zhang et al. 2025). Among all tested wild rice accessions, *O. rufipogon* exhibited the highest nucleotide diversity (0.00568) (Sun et al. 2001; Yin et al. 2015), which not only reflected that this wild rice species has retained the most abundant genomic variations during long-term natural evolution, but also provided strong evidence for its status as the core ancestral species of Asian cultivated rice (*O. sativa*). Most wild rice species had negative Tajima's D values, implying that their populations have either undergone recent expansion or been subjected to positive natural selection. In contrast, *O. barthii* and *O. glumipatula* had Tajima's D values close to zero or slightly positive, indicating that their populations are near neutral evolutionary equilibrium or maintain the polymorphism of key loci under balancing selection. However, *indica* showed a significantly positive Tajima's D value, which is a typical characteristic arising from the combination of intense artificial selection and the domestication bottleneck effect, with its evolutionary trajectory entirely driven by human breeding demands. *O. rufipogon* had the minimum FST value with *O. sativa*, and the low genetic differentiation and close genetic relationship between the two further confirmed the status of *O. rufipogon* as the direct ancestral species of *O. sativa*. The rich genetic polymorphism of wild rice furnished a sufficient genetic basis for humans to select favorable agronomic traits at the initial stage of rice domestication. We also detected that certain nsSNPs in selected genes were conserved between *O. meridionalis* and *O. rufipogon,* but appeared in the cultivated rice, implying that these variants might potentially alter the functions of the target genes. In rice, Tan et al. found that two SNPs in the coding region of PROG1 disrupt protein function, leading to prostrate growth and reduced grain yield (Tan et al. 2008). This mutation was further confirmed to be artificially selected and fixed during rice domestication (Jin et al. 2008).

Crop domestication is one of the most pivotal events that initiated human civilization. *O. longistaminata* holds a large potential for tolerance to abiotic stresses including heat, drought and salinity (Tong et al. 2023). *O. rufipogon* contains abundant beneficial genes, such as high yield and stress resistance (Zheng and Ge 2010). In this study, we found that the genes selected during the evolution of cultivated rice are mainly related to stress responses, including nitrogen compound response and temperature stimulation response. This may be related to the wide distribution range of wild rice and the complex ecological conditions (Lian et al. 2023). However, numerous valuable genes were lost throughout the process of evolution. More than 40% of the genes in the AA genome wild rice were lost in the process of evolution. Among these lost genes, many were associated with various desirable traits in rice and crucial for its growth, development, and adaptation to the environment (He et al. 2006; Zha et al. 2009; Liu et al. 2021). Wild relatives of crops typically possess a broader genetic diversity than cultivars, and the incorporation of genes from these related species in plant breeding led to a 30% enhancement in crop yields during the late twentieth century (Pimentel et al. 1997).

The growth and development of rice are often affected by various biotic stresses (plant diseases and insect pests) and abiotic stresses (drought, salinity, extreme temperatures, etc.). Resistance Gene Analogs (RGAs) are a class of gene sequences in plant genomes that possess conserved domains similar to those of known resistance genes (R genes). They may be involved in plant disease resistance defense responses, serving as core candidate resources for mining plant disease resistance genes and deciphering disease resistance mechanisms. Guo et al. employed the RGAugury pipeline and identified an average of 1,710 RGAs in *O. rufipogon*, along with a receptor-like kinase (RLK) gene *LOC_Os07g35680*, which acts as a negative regulatory factor of rice blast disease mediated by *OsMADS26*. In this study, the same method was used to identify RGAs in the pangenomes of five wild rice species. A total of 2001 RGAs were detected in the pangenome of *O. rufipogon* (Table S18). Further analysis of *LOC_Os07g35680* revealed that this gene exists in the other four wild rice species but is absent in *O. glumipatula*, implying that the gene has been lost in O.glumipatula (Table S19). The salinization and sodification of agricultural land are increasing due to the impact of climate change, water shortages, and unsustainable farming practices (Trejo-Téllez 2023). Salinity stress progressively reduces plant growth and productivity, while plants have developed complex signaling pathways to confront salt stress. However, only a few genetic variants have been identified to mediate salt tolerance in the major crop rice, and the molecular mechanism remains poorly understood (Yu et al. 2023). It is evident to us that the current strategy of rice breeding aimed at enhancing Na^+^ exclusion from uptake has reached a plateau after several decades, thereby limiting further advancements in salinity tolerance traits within this species (Fornasiero et al. 2022). Incorporating salinity tolerance traits from wild species into cultivated rice holds promise as a viable approach for improving salinity tolerance in rice (Fornasiero et al. 2022). Huang et al. report the haplotype-resolved gapless genome assembly and annotation of *O. rufipogon*, three salt-tolerant QTLs were identified through QTL mapping(Huang et al. 2024). In this study, by integrating differential expression data, a total of 42 salt stress tolerant candidate genes were identified in the selected gene set of *O. sativa*, while 32 salt stress tolerant candidate genes were identified in the lost gene set of *O. sativa*. From these, we selected the transcription factor gene *WRKY67* for functional validation. As an important member of rice gene family, WRKY transcription factor family can specifically bind to W-box in the promoter region and directly or indirectly regulate disease resistance, cold and heat, drought, high salt, senescence and other related genes, thus participating in the process of biotic and abiotic stresses, hormone signal transduction, development and metabolism in rice (Jiang et al. 2017). A modulated expression of WRKY67 alters the defense response to rice blast fungus and bacterial blight, as well as plant growth. (Vo et al. 2018; Liu et al. 2018). The RT-qPCR results confirmed that *WRKY67* and *WRKY28* had similar expression trends in the leaves and roots of cultivated rice under salt stress. *WRKY28* had been found to activate the expression of *DREB1B* by directly combining with the promoter to regulate rice salt tolerance, and the salt tolerance of *WRKY28*-overexpression rice line was enhanced while the *WRKY28*-knockout mutant was salt sensitive (Zhang et al. 2022b). Genes with similar expression patterns, functionally closely related, or members of the same signal pathway or process are likely to be closely co-regulated (Silva-Vignato et al. 2019). Under salt stress treatment with 100 mM and 200 mM concentrations, WT exhibited better growth performance than the *WRKY67-knockout* mutants. All these results indicated that *WRKY67* was a novel gene related to salt stress tolerance of rice.

In summary, the pan-genomes of 5 AA genome wild rice species were developed by using genome re-sequencing data of 180 wild rice varieties, and the genomic variation between wild and cultivated rice was detected. The results implied a potential evolutionary trend with gene flow primarily from *O. meridionalis* to *O. rufipogon* and then to *O. sativa*. Many genes related to stress response and growth regulation were identified in lost and selected genomic regions, among which *WRKY67* gene had emerged as a novel salt tolerant gene in rice.

## Materials and methods

### Data collection

The genome resequencing data of 189 wild rice varieties (180 AA genome wild rice and 9 *O. meyeriana*) were downloaded from SRA database (https://www.ncbi.nlm.nih.gov/sra/). *O. rufipogon*, the direct ancestral species of cultivated rice, has the widest natural distribution range and serves as a core material for studies on rice origin and domestication. To ensure comprehensive capture of the genetic diversity of this species, its sample size was increased to 89 accessions, which were collected from 11 countries and basically cover its natural growth range. For the other four AA genome wild rice species, we selected 20 to 26 samples respectively, and these samples comprehensively covered their core geographical distribution areas (Table S1). In addition the genome resequencing data of 39 *O. sativa* rice varieties were downloaded from rice SNP-seek database (https://snp-seek.irri.org/index.zul). Among the 39 cultivated rice accessions, 2 were *Aus/boro*, 1 was *Basmati/sadri*, 19 were *indica*, 9 were *Japonica*, 6 were *Temperate japonica*, and 2 were *Tropical japonica*. These accessions were collected from 8 countries, which ensured the representativeness of genetic diversity in cultivated rice (Table S2). All downloaded data come from the ILLUMINA sequencing platform. *Nipponbare* reference genome was downloaded from RAP-DB database (https://rapdb.dna.affrc.go.jp/).

### Construction of pan-genome

The pan-genome of wild rice was constructed by iterative assembly approach. The original sequencing data low quality and unknown base sequences(N) were checked and trimmed by using fastqc and trimmomatic tools. Bowtie2 (version 2.2.6, parameters: –I 0 –X 1000) and SAMTOOLS (default parameters) tools were then used to map the clean sequencing data to the reference genome and sort sequencing data respectively. Using MASURCA (parameters: EXTEND_JUMP_READS = 0, GRAPH_KMER_SIZE = auto, USE_LINKING_MATES = 0, LHE_COVERAGE = 25, MEGA_READS_ONE_PASS = 0, LIMIT_JUMP_COVERAGE = 300, CLOSE_GAPS = 0, CA_PARAMETERS = cgwErrorRate = 0.15, KMER_COUNT_THRESHOLD = 1, NUM_THREADS = 16, JF_SIZE = 3,500,000,000, DO_HOMOPOLYMER_TRIM = 0, SOAP_ASSEMBLY = 0) the unmapped sequences were de-novo assembled. CD-HIT (parameters: –d 0 –c 0.9 –T 16 –M 8000) removed the redundant sequences and BLASTN (parameters: -evalue 0.001) was used to remove the contaminant sequences by comparing with NCBI non-redundant nucleotide databases and finally the short contigs whose length less than 500 bp were filtered out. The final contigs sequence were then combined with the reference sequence to obtain the pan-genome sequence. The final assembled sequence quality of the pan-genome was assessed for completeness using BUSCO with the default parameters, and the eukaryote dataset as a predictive gene set for evaluation (Simão et al. 2015). Finally, MAKER was used to annotate and predict the genes in the non-reference contigs in each genome (Holt and Yandell 2011).

### Variant discovery and annotation

The wild rice varieties whole genome sequence reads of 189 accessions were first quality checked by using Fastqc and trimmed the low quality reads with Trimmomatic(Bolger et al. 2014). Secondly, Bowtie2 (version 2.2.6, parameters: –I 0 –X 1000) was used to align the trimmed reads to the *Nipponbare* reference genome, then the aligned reads were sorted by using samtools, and GATK MarkDuplicates tool removed the duplicate reads. Third, GATK HaplotypeCaller tool was used to detect the variants and GATK Variant Filtration tool to filter out low quality variants based on the default GATK recommended parameters (QD < 2.0, FS > 200, SOR > 10, MQ < 40, MQRankSum < – 12.5, ReadPosRankSum < − 8.0, DP > 3). Finally, SnpEff tool was used to annotate the SNPs(Cingolani et al. 2012). Subsequently we extracted the variants in the evolutionary selected genes from the whole genome variants of these 188 rice varieties. Structural variation was also detected by using Synteny and Rearrangement Identifier (SyRI) tool (Goel et al. 2019).

### Phylogenetic analysis

Single-nucleotide polymorphism (SNP) loci with a minor allele frequency (MAF) < 0.01 and a genotype missing rate > 0.2 were filtered out using PLINK (parameters: –maf 0.01, –geno 0.2). The retained population SNP dataset was subsequently used as the input for phylogenetic analysis, and a population phylogenetic tree of the 227 rice accessions was constructed using IQ-TREE. (Minh et al. 2020). Then the online tvBOT tool (https://www.chiplot.online/tvbot.html) was used to visualize and beautify the phylogenetic tree (Xie et al. 2023). Build PAC with GCTA tool (Yang et al. 2011). Population structure analysis was conducted using ADMIXTURE tool. In order to obtain the best estimated K, we calculated cross validation (CV) values for K = 1 to K = 15, and we selected the K value with the minimum CV value. Finally, the population structure diagram with optimal classification K value was plotted in R version 4.3.

### ABBA-BABA test

We used ABBA-BABA test to infer gene flow and genomic introgression among the genomic data. Low frequency SNP sites were filtered out by using PLINK software based on this option (–maf 0.01, –geno 0.2, –allow-extra-chr). *Brachypodium distachyon* was selected as outgroup, *O. sativa* as P1, *O. rufipogon* as P2, and other wild rice as P3. After preparing the sample grouping information table, D statistics were calculated using Dsuite tool (Malinsky et al. 2021).

### Nucleotide diversity and selective scanning

Using VCFTOOLS we calculated population nucleotide diversity (π) value, Tajima'sD value, genetic differentiation index (FST) with a sliding window length of 40 KB and sliding step of 10 kb. XP-CLR (cross-population composite likelihood ratio) statistical analysis was also performed according to the method in Su et al. study (Su et al. 2018). A sliding window with FST values greater than the 95th percentile of genome-wide FST values and XP-CLR statistics greater than the 95th percentile of genome-wide were identified. Finally, the overlapped regions of the two-part approaches were considered as the final selected regions.

### Gene presence and absence variation (PAV) analysis

Detection of PAV gene in the pangenomes was conducted using SGSGeneLoss tool with the parameters settings of minCov = 2, lostCutoff = 0.05 and covCats = 0,2,5,10,20 (Golicz et al. 2015).

### Pan-genome and protein–protein interaction network

Protein–protein interaction network data for five AA genome wild rice was downloaded from the STRING (https://cn.string-db.org/) database. Since the protein–protein interaction network data of *O. longistaminata* could not be found in the STRING database, the protein–protein interaction network data of *O. sativa* were downloaded. All protein sequences of *O. longistaminata* pan-genome were aligned with protein sequences of *O. sativa* by using BLASTp software. If the aligned protein of *O. sativa* was in the interaction network, it was considered that the protein of *O. longistaminata* pan-genome matched with it was also in the protein–protein interaction network.

### Identification of syntenic genes

Homology analysis using OrthoMCL (Li et al. 2003). Low-quality protein sequences with shorter length or containing more stop codons were removed. BLASTP alignment of all protein sequences to each other (All-vs-All BLASTP). BLAST database was constructed by using all protein sequences, and BLASTP alignment was performed on all sequences and database, and alignment results with E-value less than 1e-5 were selected. Alignment results with coverage less than 50% were filtered out.

### Plant growing and salinity stress

The transcriptome data of *O.rufipogon* treated with salinity stress was obtained from the study of Zhou et al. The salinity stress treatment conditions of *Nipponbare* were consistent with *O. rufipogon* (Zhou et al. 2016). *O.rufipogon* samples had no biological replicates. *Nipponbare* rice seed was immersed in distilled water in dark room, and the uniformly germinated seeds were sown in 96-well plates supported by a plastic container. The seeds were then grown in the artificial climate chamber, the growth medium was updated every 3 days. After 14 days, the salinity stress was conducted by treating the *Nipponbare* seedlings with 100 mM and 200 mM NaCl medium for 3 days. Subsequently, the leaf and root samples were collected and immediately frozen in liquid nitrogen. For RNA extraction, 10 plants were collected and mixed to minimize the effect of transcriptome unevenness among different plants. Three biological repeats were taken from each group of samples and transcriptome sequencing was performed on an Illumina Hi-seq 2000 Sequencer. The sequence reads were submitted to SRA database (BioProject: PRJNA1103978).

### Transcript profiling of wild and cultivated rice under salinity stress

To reveal the expression patterns of select and lost genes in the cultivated and wild rice under abiotic stresses, we utilized transcriptome data of wild rice under salt stresses from the GenBank GEO dataset (https://www.ncbi.nlm.nih.gov/gds/) (Accession: GSE73181). *Nipponbare* genome and *O. rufipogon* pan-genome were used as a reference for alignment and gene quantification. The quality check of the raw reads was done by using Fastqc and the low-quality reads were trimmed using Trimmomatic (Bolger et al. 2014). The Hisat2 was used for sequence alignment to *Nipponbare* (Kim et al. 2015), the StringTie assembler was used to assemble and quantify the reads (Pertea et al. 2015). The DEseq2 was used to identify differentially expressed genes (DEGs) (FPKM ≥ 0.5, |log2fold-change|≥ 1 and P-value ≤ 0.05).

### RT-qPCR analysis

Total RNA was extracted from rice leaves and roots using the MiniBEST Plant RNA Extraction Kit (TaKaRa), and cDNA was synthesized using the HRbio™ III 1st Strand cDNA Synthesis SuperMix for qPCR (OneStep gDNA Removal). Real-time quantitative PCR (qRT-PCR) was performed on a 96-well plate using HRbio™ qPCR SYBR Green Master Mix (Low Rox Plus). The rice actin gene was used as the reference gene to normalize the target gene expression, which was calculated using the relative quantization method △△Ct (Livak and Schmittgen 2001). Primers are shown in Table S3. Each reaction was performed with four technical replicates and four biological replicates.

### Subcellular localization

The stem segment of two-week-old rice seedlings were taken and cut into thin slices of 0.5 mm. The cut slices were transferred to a conical flask containing 50 ml of enzymatic solution. It was wrapped with tin foil paper, vacuumed for 30 min, then transferred to a horizontal constant temperature shaker at 60 rpm, 25 °C conditions, and enzymolyze for 4 h. Using the prepared W5 solution to stop the enzymatic hydrolysis, and the released protoplasts were collected in a 50 ml centrifuge tube on a constant temperature oscillator (200 rpm, 40 min) until the color of the W5 solution gradually becomes transparent. The prepared K3 solution was used to separate the collected protoplasts and debris. After separation, an appropriate amount of W5 solution were added for washing, then centrifuged at 100 g, 4 °C, acceleration 1 and deceleration 0, for 3 min, and re-suspend the protoplasts in 5 ml of W5 solution. After recovery on ice for 1 h, microscopy was performed. Based on the microscopic results, the amount of extracted protoplasts was calculated, and the final concentration was adjusted to 2.5–3 × 10^6^ cells/ml with MMG solution. In a 2 ml centrifuge tube, 10 μl of plasmid was added at a concentration of 1–1.5 μg/μl and 200 μl of protoplasts, and gently mixed. Then 220 μl of PEG solution was added and gently mixed, and let stay at room temperature in the dark for 5 min. Then 1000 μl of W5 solution was added to stop the reaction. Afterwards, it was centrifuged at 300*g*, 4 °C, acceleration 5 and deceleration 1, for 3 min. the supernatant was removed, 220 μl of W1 solution was added, and it was incubated overnight for 16 h. Finally, it was observed under a laser confocal microscope by gently resuspending the incubated protoplasts, taking 6 μl to a slide, placing it on the LEICA SP8 laser confocal stage, and observed under excitation light at 488 nm and absorption spectrum of 510–550 nm.

### Phenotypic analysis of salt stress in WKRY67 mutant’s rice

The sterilized rice varieties Zhonghua11 (ZH11) and *WKRY67* mutant rice seeds were soaked in water at 37 °C for 7 days, then the germinated seeds were placed in nutrient soil and grown in a culture box under a short-day photoperiod cycle of 25 °C/16 °C (light/night) with 8 h of light and 16 h of night. Two weeks after cultivation, the rice seedlings were treated with four different concentrations of NaCl (0/100/200/300 mM) added to the nutrient solution. Finally, after 5 days of salt stress, the growth of rice seedlings was observed.

## Supplementary Information

Additional file 1.

Additional file 2.

## Data Availability

The datasets analyzed in this study are available in the GEO database (accession: GSE73181) and SRA database (BioProject: PRJNA1103978). All data that support the findings in this study are available in this article and its supplementary files.
