# Supplementary material for: Pan-genome of wild and cultivated rice uncovers genetic diversity, lost and selected sequences during rice domestication
Source: Rice (N Y). 2026 Feb 24;19:21. doi: 10.1186/s12284-026-00893-w (PMC13035952; doi:10.1186/s12284-026-00893-w)
Supplement: Supplementary file 1 — Additional file 1. [file 12284_2026_893_MOESM1_ESM.pdf]

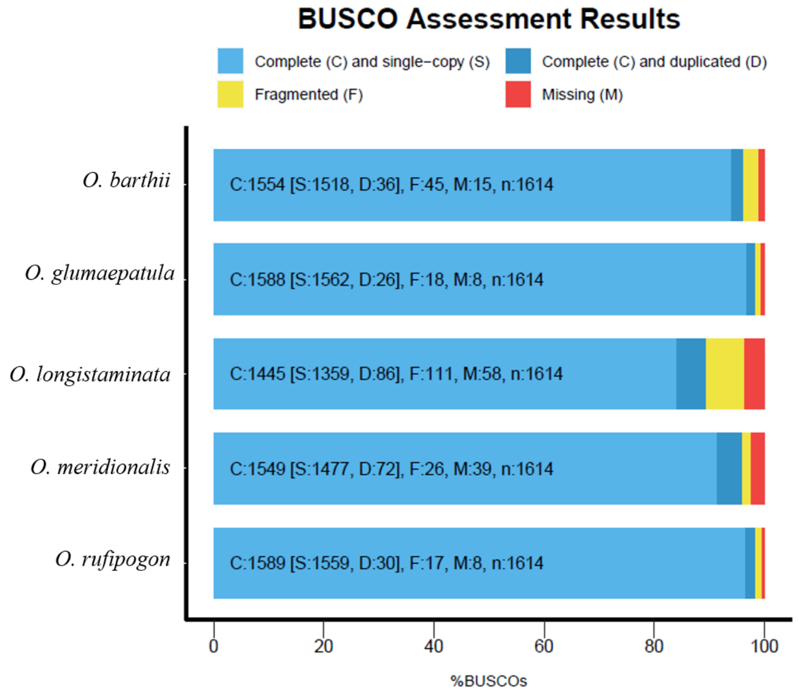

Figure S1. Integrity of wild rice pan-genome by BUSCO

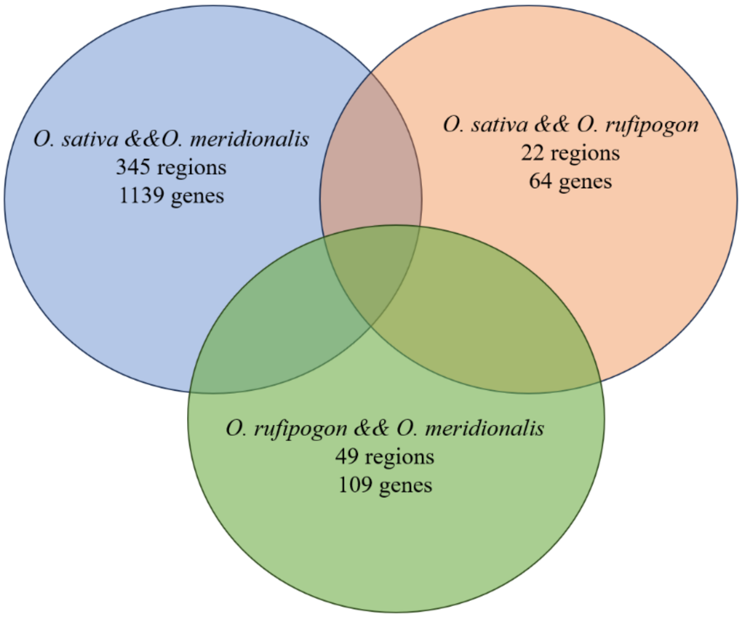

Figure S2 The number of selected regions and genes in the evolution of wild to cultivated rice

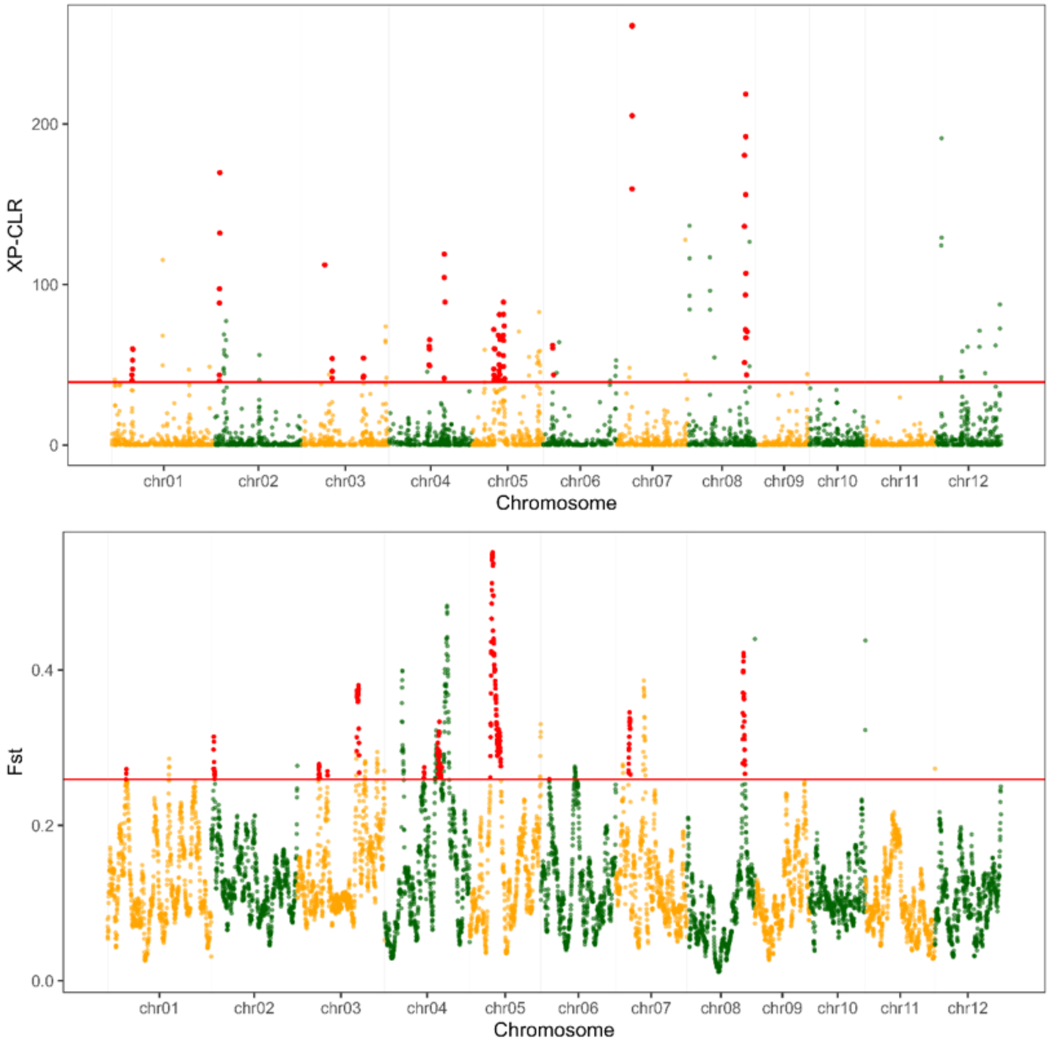

Figure S3. Selected regions in the evolution of *O. rufipogon* to *O. sativa*. The red line is the threshold for the top 5%, and the red dot indicates the selected regions

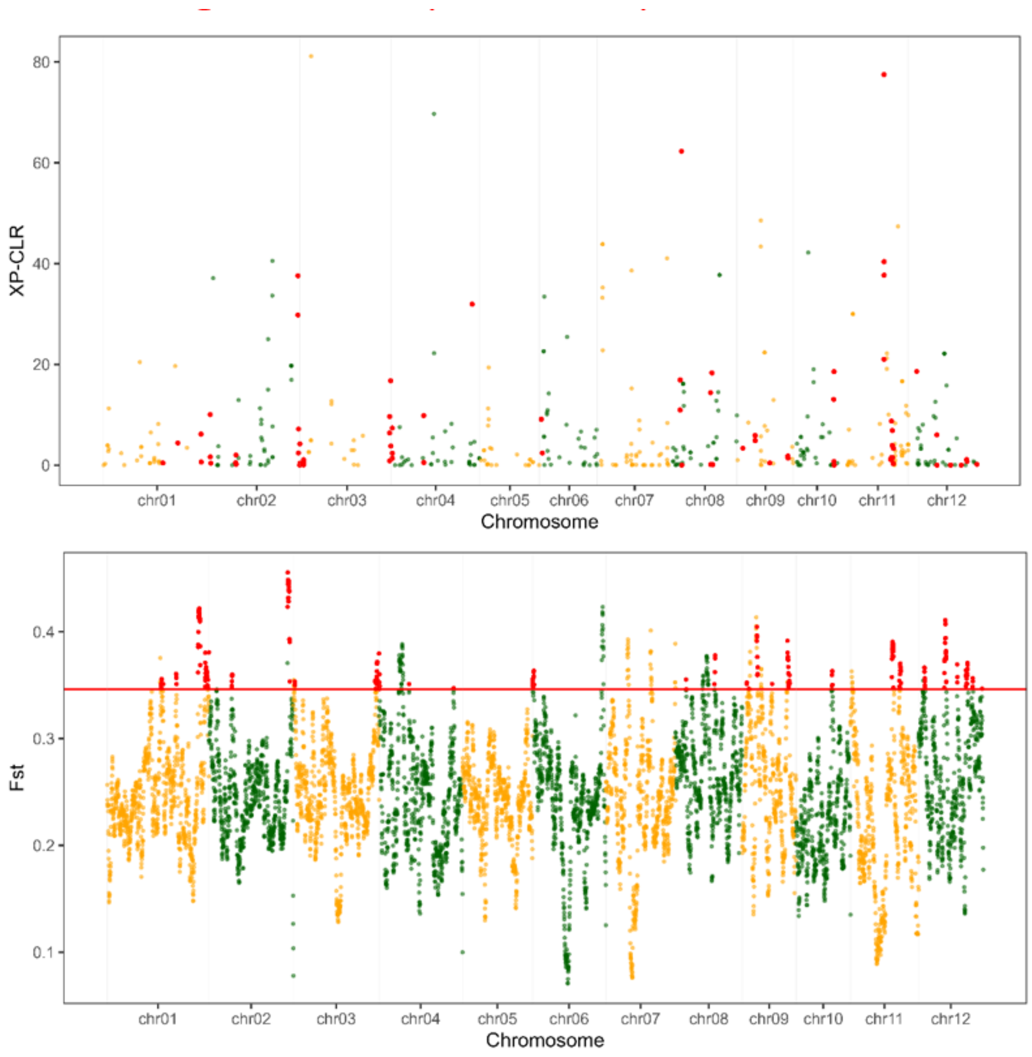

Figure S4. Selected regions in the evolution of *O. meridionalis* to *O. rufipogon.* the red line indicates the threshold for the top 5%, and the red dot indicates the selected regions. Because of the region of XP-CLR value>0 is in the top 5% of the whole genome, so no threshold had been set for XP-CLR value.

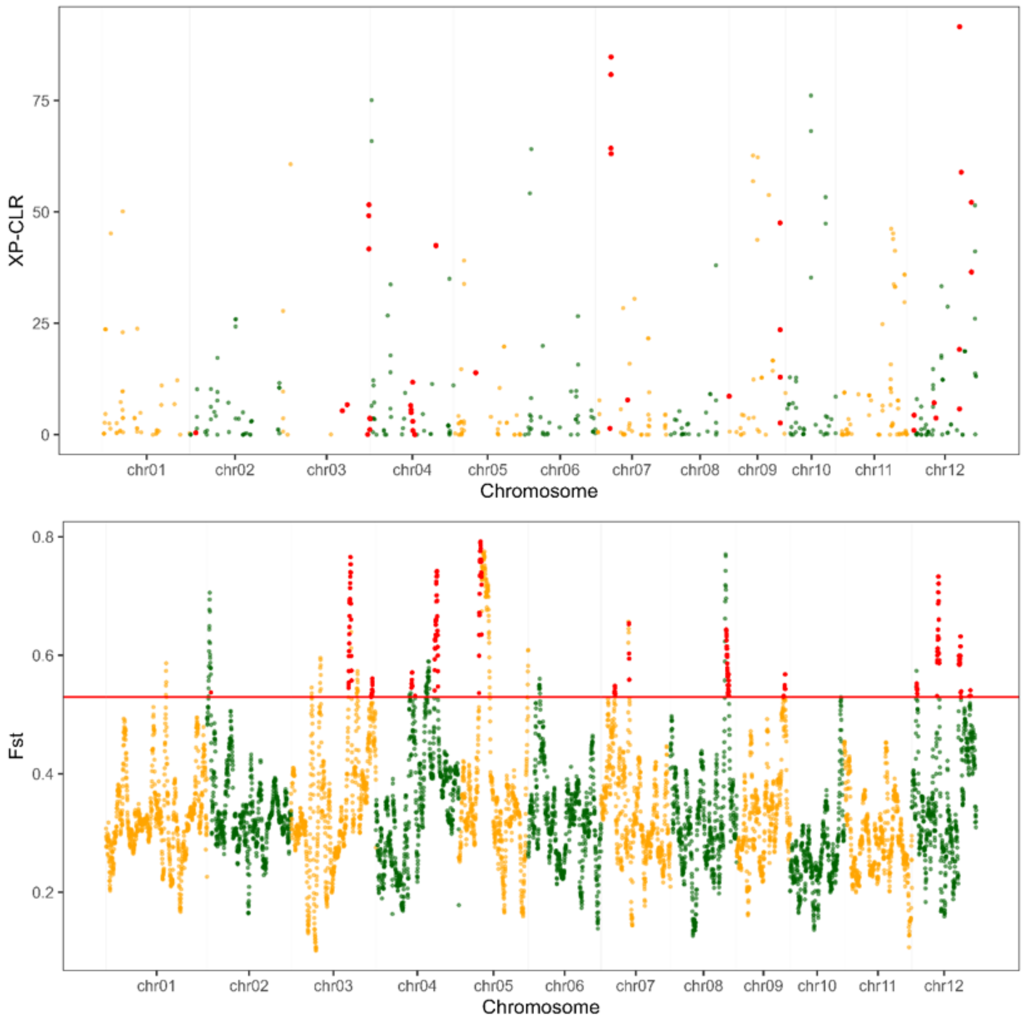

Figure S5. Selected regions in the evolution of *O. meridionalis* to *O. sativa.* The red line is the threshold for the top 5%, and the red dot indicates the selected regions. Because of the region of XP-CLR value>0 is in the top 5% of the whole genome, so no threshold had been set for XP-CLR value.

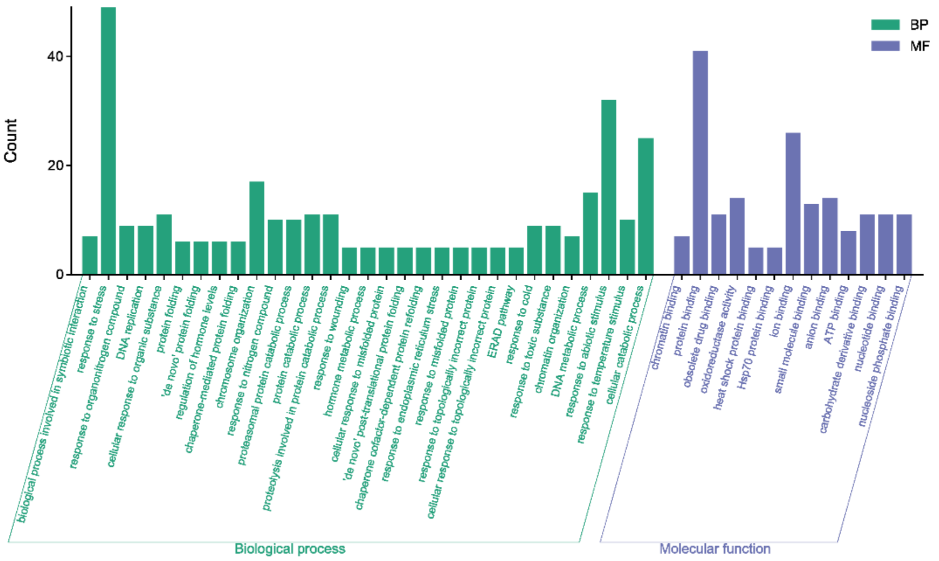

Figure S6. GO enrichment analysis of selected gene sets in the evolution of *O. meridionalis* to *O. rufipogon*.

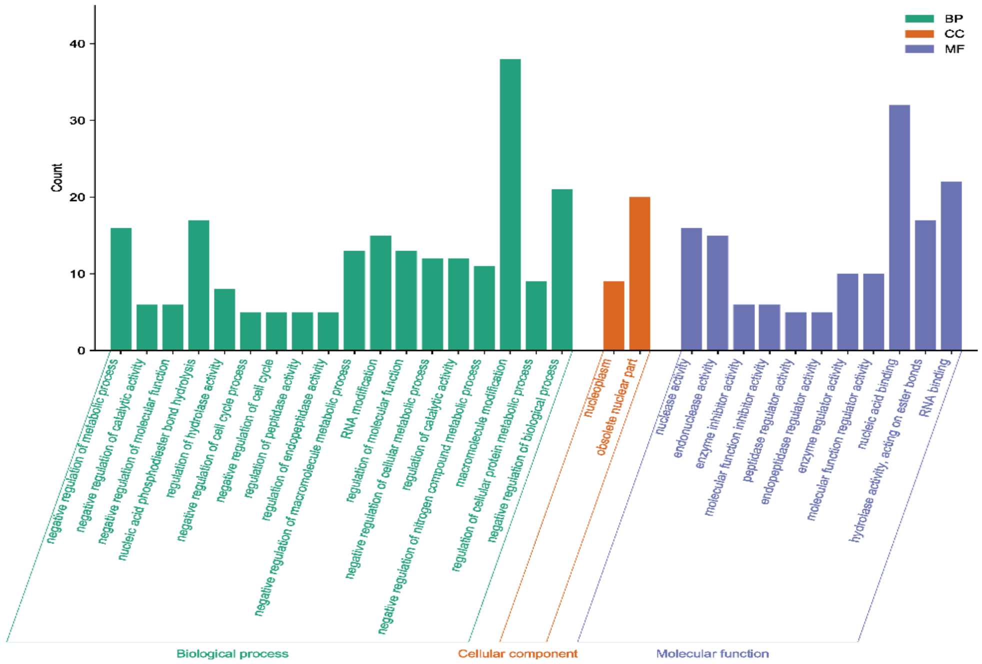

Figure S7. GO enrichment analysis of the genes in the selected regions in the evolution of *O. rufipogon* to *O. sativa*

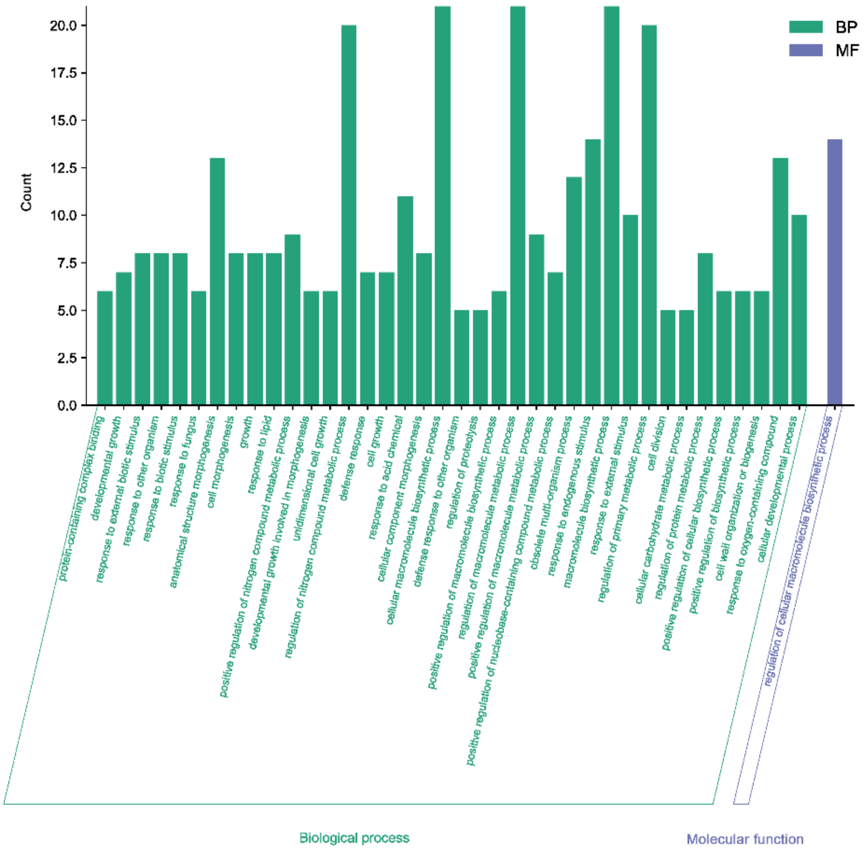

Figure S8. GO enrichment analysis of selected gene sets in the evolution of *O. meridionalis* to *O. sativa*
